# 1352. Changing Quality Indicators by Monitoring Veterans Using the Sexually TRansmitted Infection Key Evaluation (STRIKE) Dashboard

**DOI:** 10.1093/ofid/ofab466.1544

**Published:** 2021-12-04

**Authors:** Minh Q Ho, Linda Chia, Matthew Cole, Tho Nguyen, Karen Slazinski

**Affiliations:** 1 Orlando VA Healthcare System, 14014 Deep Forest Court, Florida; 2 VA, Bellevue, Washington; 3 VA Capital Health Care Network (VISN 5), Veterans Health Administration, Huntsville, Alabama; 4 Orlando VA HCS, Orlando, Florida

## Abstract

**Background:**

During the COVID-19 pandemic, there have been multiple reports concerning patients falling out of healthcare. The National VA HIV and Hepatitis and Related Conditions (HHRC) has created the Sexually TRansmitted Infection Key Evaluation (STRIKE) Dashboard to help clinicians identify Veterans who need to complete co-testing for sexually transmitted infections (STIs) or human immunodeficiency virus (HIV) and allows providers to document if the Veteran was offered pre-exposure prophylaxis (PrEP).

STRIKE Interface Screen

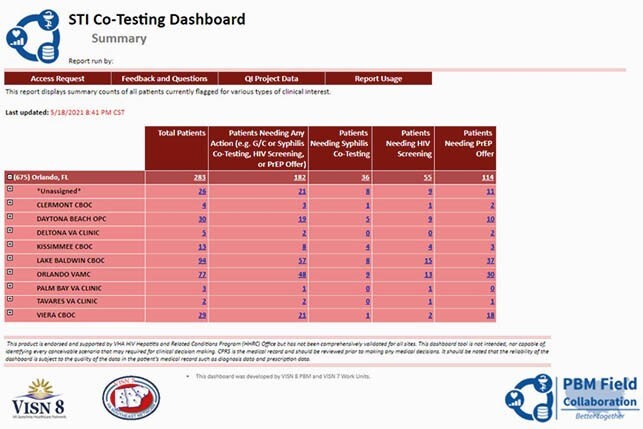

**Methods:**

A national VA Veteran dataset was generated from data within the Corporate Data Warehouse (CDW) that included all active PLWH. Positive HIV status is evaluated based on positive antibody test and positive confirmatory result or positive viral load lab result. Negative HIV status is evaluated based on a negative antibody test in the past year. Of the 140 sites, 39 participated but only 9 were active throughout the period of October 1, 2020 to March 31, 2021. Active and nonactive participating sites had metrics assessed across the study period at 3 time points: October 1, 2020, January 1, 2021 and April 1, 2021. Sites with at least 48 visits to report across the 6-month QI period were considered active.

Patient level data for review

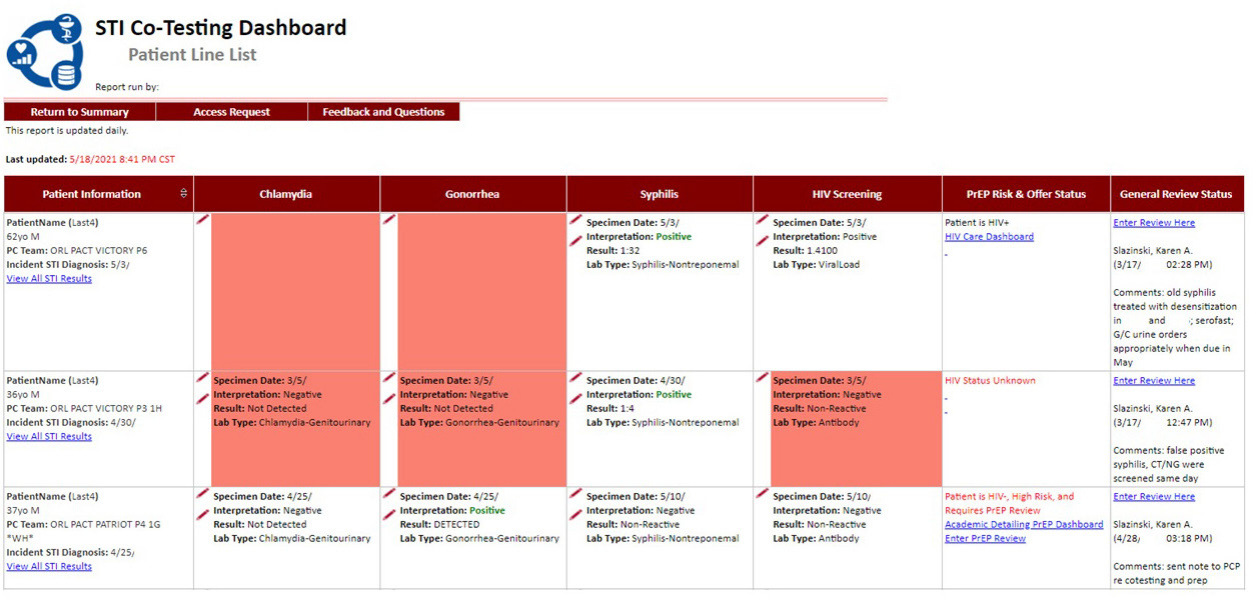

Additional patient level data for review

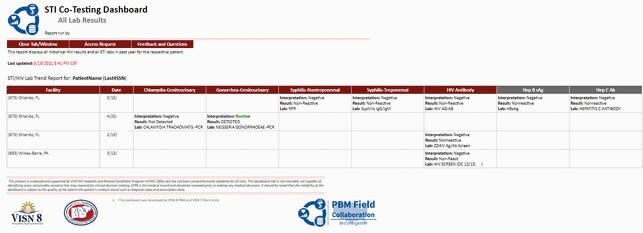

**Results:**

Multiple sites had scarcity of supplies due to the national shortage of CT/NG Test Kits during COVID-19. To improve access to CT/NG testing, the dashboard suppress the list of Veterans with + syphlis who were not co-tested for CT/NG. Co-testing improved from 60.2% to 77.2% in active sites and from 61.9% to 68.7% for nonactive across the study period. Percent of Veterans with completed HIV testing on or after STI diagnosis in active sites had an upward trend of 2.1% compared to the nonactive which increased 0.6%. Likewise, new diagnosis of STI for those on PrEP increased 2.6% in active and 0.5% increase in nonactive. On average, active sites increased percent of high risk veterans with active PrEP prescriptions by 2%, compared with nonactive that only increased 1%.

STRIKE Co Testing and HIV Screen performed

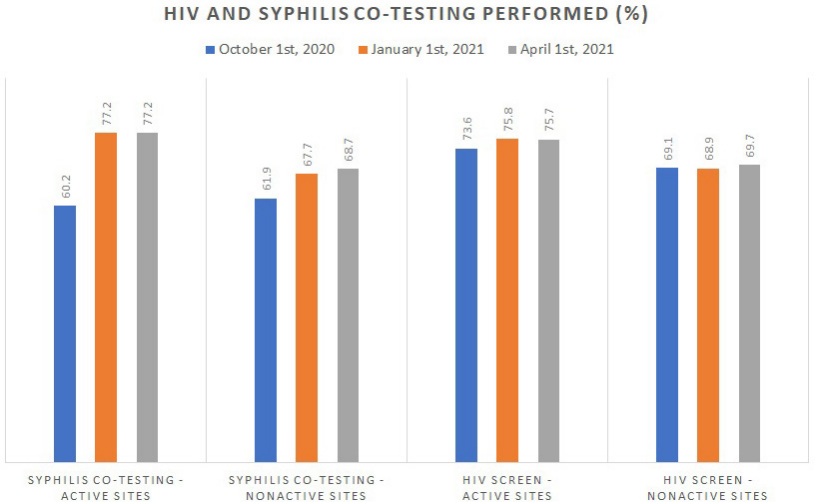

This graph shows average of Syphilis Co Testing and HIV screen performed by active and nonactive sites as measured at three different time point.

Number of Veterans on PrEP with STIs

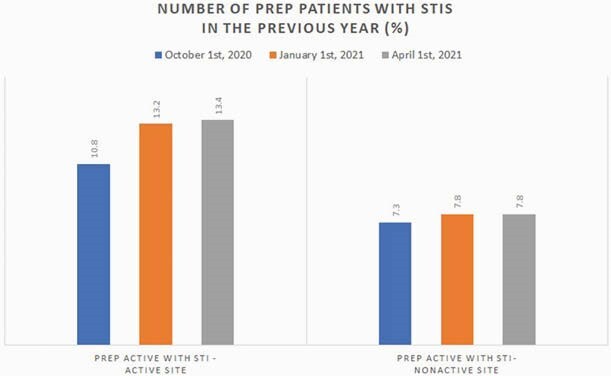

This graph shows the average percent of veterans on PrEP with STIs between active versus nonactive sites as measured at three time points

Active PrEP Prescriptions Across All Sites

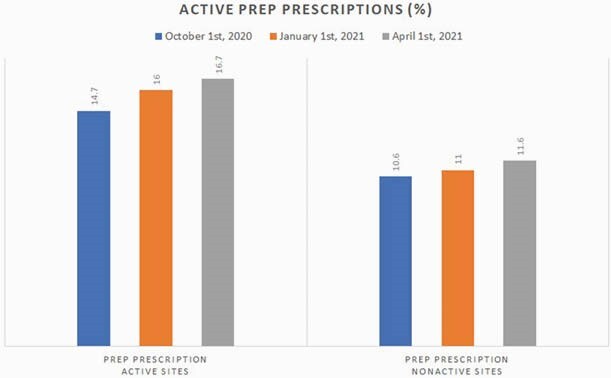

This graph shows average percent of active PrEP prescriptions for high risk patients between active versus nonactive sites as measured at three time points

**Conclusion:**

The STRIKE Dashboard efficiently flags Veterans with STI diagnosis who need completion of STI co-testing, including HIV testing and PrEP offer. Active participating facilities who used the STRIKE Dashboard improved STI Co-testing and PrEP prescription in a short 6 month period even during COVID-19.

**Disclosures:**

**All Authors**: No reported disclosures

